# Enhancing Activity in the Right Temporoparietal Junction Modifies the Effect of a High CEO-to-Employee Pay Ratio on the Perceived Investment Potential in the Construction Industry

**DOI:** 10.3389/fnins.2022.872979

**Published:** 2022-05-12

**Authors:** Xiaolan Yang, Jiaqi Wang, Shu Chen

**Affiliations:** School of Business and Management, Shanghai International Studies University, Shanghai, China

**Keywords:** CEO-to-employee pay ratio, perceived investment potential, construction industry, right temporoparietal junction, transcranial direct current stimulation

## Abstract

As an increasing number of governments require the disclosure of companies’ compensation information, compensation management is becoming an important part of internal management in the construction industry. Although the literature has shown that disclosing a high CEO-to-employee pay ratio will cause various effects on the decision-making of a company’s potential investors, there is little evidence on the neural basis of such effects. Given that previous neuroscience studies have shown that the right temporoparietal junction (TPJ) is associated with altruistic behaviors, this study used transcranial direct current stimulation (tDCS) to explore the role of the right TPJ in the effects of the CEO-to-employee pay ratio on potential investors’ perceived investment potential in the construction industry. The results show that enhancing activity in the right TPJ significantly reduced the perceived investment potential of female participants, especially those with no investment experience, when the company’s CEO-to-employee pay ratio is high compared to when the pay ratio is medium. This effect was not observed in male participants. The mechanisms underlying these effects of tDCS in the right TPJ on the perceived investment potential were also explored. The main contribution of this study lies in its pioneering exploration of the neural basis of investment decision-making regarding the CEO-to-employee pay ratio. Additionally, it reveals individual feature-based differences in the role of the TPJ in investment decision-making and its possible mechanisms.

## Introduction

As an increasing number of governments require the disclosure of companies’ compensation information, compensation management has become an important research topic in recent years. For instance, the Dodd-Frank Act of the United States stipulates that listed companies must disclose the compensation of their CEO, the median compensation of all employees (except the CEO) and the ratio between the two. In the construction industry, compensation management is a considerable part of internal management. To make project management convenient, it is quite common for large construction companies to have many subsidiary companies. Thus, decisions regarding the compensation of the CEO (either parent or subsidiary) and other employees have to be made frequently. With the mandatory disclosure of compensation information, these decisions increasingly impact the efficiency and success of a construction company’s business.

Previous studies have shown that disclosing a high CEO-to-employee pay ratio affects the decision-making of a company’s potential investors. It has been found that investors believe that incorporating the CEO-to-employee pay gap into investment decisions can improve investment returns (Barton and Mercer, 2005). Kelly and Seow (2016) used experiments to demonstrate that disclosing a higher-than-industry pay ratio has a significant indirect negative effect on perceived investment potential through perceived CEO pay fairness. Based on surveys of stakeholder groups, Arnold and Grasser (2018) found that investors care about fairness and that there is capital market outrage toward high amounts of CEO compensation. Atan et al. (2018) found that a high CEO-to-employee pay ratio usually represents a poor compensation management situation and causes investors to have a negative impression when evaluating a company. When investors are aware of such conditions, they ultimately show lower investment willingness (Yang et al., 2021). Furthermore, Pan et al. (2021) examined market data and found that a high CEO-to-employee pay ratio affects investors’ evaluation of a company’s operating conditions and that the negative evaluation usually has an unfavorable impact on the value of the company, with investors’ prosocial preferences acting as a moderator. The CEO-to-employee pay ratio has also been found to have interactive effects with other company features. For example, if a CEO-to-employee pay ratio that is higher than the industry level is disclosed by a company with a good reputation, the company will be punished more than companies with bad reputations; conversely, if a company with a poor reputation discloses a CEO-to-employee pay ratio that is lower than the industry level, it will obtain a higher return (Seow et al., 2019).

Nevertheless, the neural basis of the effects of the CEO-to-employee pay ratio on investment decision-making has seldom been explored. It is reasonable to believe that investment decision-making regarding compensation is probably related to altruism. A higher level of altruism may reduce people’s willingness to invest in a company that is less socially responsible, i.e., a company with a high CEO-to-employee pay ratio. Meanwhile, a higher level of altruism may also lead investors to be more aware of the negative effect of a high pay ratio on a company’s investment potential, which will speed up the reduction in their willingness to invest in the company. Previous neuroscience studies have shown that the right temporoparietal junction (TPJ) is associated with individuals’ altruistic behaviors. Morishima et al. (2012) used a functional magnetic resonance imaging (fMRI) technique and found that brain activation in the right TPJ was highest when the cost of altruistic behavior was lower than the individual’s maximum willingness to pay for altruistic behavior. Studies using transcranial direct current stimulation (tDCS) have also provided evidence on the causal relationship between right TPJ activity and altruistic behaviors. After the right TPJ was stimulated through tDCS, participants increased the amount that they allocated to charity (Li et al., 2020). Similarly, Yang et al. (2021) found that enhancing activity in the right TPJ increased participants’ altruism and their willingness to invest in socially responsible funds.

This study aims to explore how enhancing activity in the right TPJ through tDCS influences the effects of the CEO-to-employee pay ratio on investors’ perception of a company’s investment potential. According to previous studies, a higher activation level in the right TPJ results in a higher level of altruism. Therefore, this study hypothesizes that tDCS will reduce investors’ perception of the investment potential of a high-pay-ratio company compared with that of a fair-pay-ratio company. Furthermore, this study mainly focuses on investment decision-making in the construction industry since it is where the interest of the study lies and because compensation decisions are more frequent in the construction industry than some other industries. To the best of our knowledge, there is little evidence on the effects of the CEO-to-employee pay ratio on investment decision-making in the construction industry and little evidence on the neural basis of these effects.

In addition, this study pays attention to the relationship between the effects of tDCS stimulation and some individual socioeconomic features, namely, gender and investment experience. Studies have revealed that investors with different genders or levels of investment experience show differences in their investment decision-making, especially when such decision-making is related to altruism. For example, women choose to invest their financial resources more conservatively and are generally more risk averse than men (Bajtelsmit and VanDerhei, 1997; Embrey and Fox, 1997; Yuh and Hanna, 1997). Marinelli et al. (2017) investigated behavioral data obtained from the clients of an Italian bank and found significant gender differences in investment behaviors with regard to the decision-making process, risk preferences and actual portfolios. Christie (2018) found that Indian female investors were more prone to biases such as mental accounting, anchoring, availability, loss aversion, regret aversion, and representativeness. Moreover, Nilsson (2008) and Dorfleitner and Utz (2014) found that female investors were more prone to make socially responsible investments. It has also been found that female investors constitute the majority of socially responsible investors and are younger and more educated than conventional investors (Tippet and Leung, 2001; Owen and Qian, 2008; Bauer and Smeets, 2015). Investors’ investment experience also plays an important role in affecting people’s willingness to invest (Martin, 2019). Chang and Wei (2011) found that veteran investors tended to attach more importance to corporate governance information and increased the weight of such information in their investment decisions. Kelly and Tan (2017) also found that in the face of information disclosure, experienced investors were highly sensitive to the type of disclosure and the approach taken to disclose information, while novice investors had no pronounced traits in response.

In summary, this study applied tDCS to temporarily enhance the activity in the right TPJ of the participants to investigate its causal effect on the participants’ perceived investment potential of a construction company under different CEO-to-employee pay ratio scenarios. The participants who received stimulation were first given the financial and compensation information of a construction company and were then asked to report their perceived investment potential. Based on the previous literature, our hypothesis is as follows: Compared to receiving sham stimulation, participants who receive anodal stimulation of the right TPJ will become more altruistic and more aware of the negative effect of a high CEO-to-employee pay ratio on a company’s investment potential. This study further checked whether the effects of tDCS were the same for participants with different genders and levels of investment experience. Finally, this study explored the mechanisms underlying the effects of tDCS that were found.

## Materials and Methods

### Subjects

A total of 184 healthy college students (92 males, 92 females, mean age: 20.91 years, ranging from 17 to 29 years) were recruited to participate in the experiment, which was conducted in the Key Laboratory of Applied Brain and Cognitive Sciences, Shanghai International Studies University. All participants were right-handed students at Shanghai International Studies University and had no history of mental illness or neurological disorders. The participants were randomly assigned to 4 treatments with a 2 (sham/active stimulation) * 2 (medium/high CEO-to-employee pay ratio) between-subjects design: the sham-medium treatment (*n* = 46; males: 23, females: 23; mean age: 20.78), the sham-high treatment (*n* = 46; males: 23, females: 23; mean age: 20.78), the active-medium treatment (*n* = 46; males: 23, females: 23; mean age: 20.67), and the active-high treatment (*n* = 46; males: 23, females: 23; mean age: 21.65). Before the experiment, the participants were required to sign a tDCS informed consent form. In addition, the experimental design was approved by the laboratory ethics committee. The experiment lasted approximately 1 h, and each participant received a payoff of 60 RMB (approximately $9.50). After the experiment, none of the participants reported any side effects, such as headaches and dizziness. Additionally, a 5-point scale (1: very uncomfortable, 5: very comfortable) was used to measure the comfort level of the participants in the experiment (Wen et al., 2019). In general, the participants reported being comfortable (mean point: 4.17), which ensured that our experiment was generally carried out in a pleasant state.

### Transcranial Direct Current Stimulation

**FIGURE 1:**
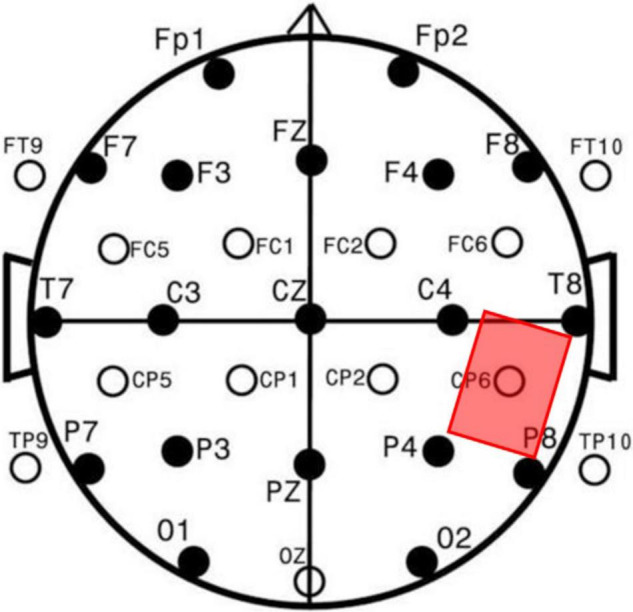
The location of the anodal electrode in the stimulation.

Transcranial direct current stimulation is a non-invasive brain stimulation technique. The stimulation equipment was manufactured by Soterix Medical Inc. (New York, NY, United States) and used two 9 V batteries to generate a constant direct current. When using the device, one of two rectangular saline-soaked sponge electrodes (size: 5 cm * 7 cm) was placed on the participant’s target brain region—the right TPJ. Based on the International 10/20 EEG Positioning System (Jasper, 1958), the center of the anodal electrode was placed over CP6 (Jurcak et al., 2007; Koessler et al., 2009). The cathodal electrode was placed on the opposite (left) side of the participant’s cheek (Hsu et al., 2011; Berryhill and Jones, 2012; Tseng et al., 2012; Mai et al., 2016; Yang et al., 2021). Figure 1 shows the position of the anodal electrode. In the case of active stimulation, the participants received a constant 1.5 mA direct current for 20 min, which was designed to induce cortical excitability in the target region without causing any physical damage to the participants. According to previous studies, anodal stimulation enhances the excitability of the target brain region (Nitsche and Paulus, 2000). For sham stimulation, the current was switched on for only 60 s, i.e., 30 s at the beginning of the 20 min and 30 s at the end of the 20 min, which has been proven reliable by previous studies (Gandiga et al., 2006).

### Experimental Design

#### Perceived Investment Potential Measurement Task

In this task, the participants were asked to carefully read some information regarding a listed company in the construction industry and to then report their perception of the company’s investment potential. The design of the materials and questions displayed to the participants referenced the study of Kelly and Seow (2016) to a large extent. The company in this task was a real American listed company (denoted as Company X in the task) classified under “construction and engineering” according to the Global Industry Classification Standard. In 2020, the company’s financial performance was in the 75% quartile of the industry. The business scope of this company mainly included the engineering and construction of energy projects, pipeline services and other construction services.

The participants were first asked to imagine that they were considering making a medium- to long-term investment in Company X. Then, they were sequentially provided a brief introduction to the company, its selected financial data from 2018 to 2020, and the compensation data of the company and its comparison group companies in 2020. The financial data included the main items of the balance sheet and income statement as well as the company’s return on equity, extracted from its real annual reports. These data generally showed that the company’s financial performance had improved in the previous 3 years. In addition, the participants were informed that the 3-year change (rise) in the price of Company X’s common stock had exceeded the 60th percentile of its comparison group companies. Meanwhile, since the participants were Chinese students, the unit of currency was changed from US dollars to RMB, but the numbers were kept unchanged. These numbers with the changed unit of currency were also reasonable for a Chinese construction company.

The compensation data included the CEO’s annual total compensation, the median annual total compensation of all employees (except the CEO), and the ratio of the CEO’s annual total compensation to the median annual total compensation of all employees (except the CEO) of Company X and the corresponding mean values of its comparison group companies (17 listed companies in the same industry) in 2020. The industry compensation data were provided because previous studies have pointed out that investors often compare the status of a company with the average level of the industry when making investment decisions (Revsine et al., 2014). The real values for these data were made public because of the Dodd-Frank Act and can be obtained from the website of the US Securities and Exchange Commission. In our experiment, the industry mean compensation data were set at their real values, and Company X’s compensation data were manipulated based on different treatments. For all treatments, the median annual total compensation of all employees (except the CEO) of Company X was manipulated to be approximately the same level as the industry mean data (Company X: 68,871, industry mean: 68,759). For the medium CEO-to-employee pay-ratio treatments, the pay ratio was manipulated to be approximately the same as the industry mean data (Company X: 81.73, industry mean: 82.53), while for the high CEO-to-employee pay-ratio treatments, the pay ratio was manipulated to be much higher than the industry mean data (Company X: 136.95, industry mean: 82.53). This typical ratio was chosen because it was the real CEO-to-employee pay ratio of a company in the industry whose pay ratio was ranked as being in approximately the 85% quartile of the comparison group companies. The corresponding annual total compensation of the CEO could be calculated based on the median annual total compensation of all employees (except the CEO) and the CEO-to-employee pay ratio (medium pay-ratio treatments: 5,628,827, high pay-ratio treatments: 9,431,883, industry mean: 5,674,940). In addition, the unit of currency was again changed from US dollars to RMB. These numbers with the changed unit of currency were also reasonable for a Chinese construction company.

#### Equity Sensitivity Measurement Task

Referring to Kelly and Seow (2016), this study used the equity sensitivity instrument from Huseman et al. (1985) to measure and control for the participants’ differential reactions to perceived inequity. The task contains five questions, and for each question, the participants were asked to divide 10 points between two choices: One choice placed more emphasis on one’s outcome, and the other placed more emphasis on one’s input. The total points of the choices placing more emphasis on one’s input in the five questions were summed. The theoretical value of the sum range was 0–50 points, and the higher the score was, the stronger the participant’s preference for one’s input to be greater than one’s outcome.

#### Risk Preference Measurement Task

This task was used to measure and control for the participants’ risk preference, which plays an important role in investment decision-making. The study adopted the method of Falk et al. (2018), which consisted of two parts. In the first part, the participants were asked to answer the following question on a 10-point scale: “In general, how willing or unwilling are you to take risks?”; on this scale, 0 means “completely unwilling to take risks,” and 10 means “very willing to take risks.” The second part consisted of five multiple-choice questions. There were two choices for each question, and the participants had to make their decision between “A draw with an equal chance of receiving 300 RMB or receiving nothing” and “A sure payment of some amount (the amount varies based on the participant’s previous decisions).” The risk preference of the participants could be calculated based on their answers in the two parts.

#### Procedure

**FIGURE 2:**
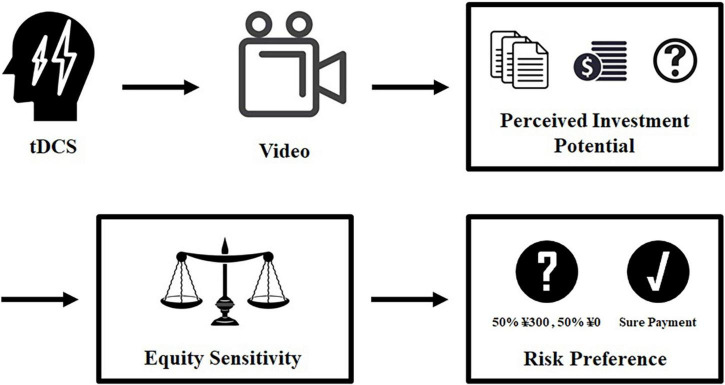
The flow chart of the experimental procedure.

The experimental tasks were programmed and realized *via* the oTree platform (Chen et al., 2016). After entering the laboratory, the participants were randomly seated and read and signed the tDCS experiment informed consent form. Afterward, the participants were asked to maintain a comfortable posture, the tDCS device was placed on their head, and then 20 min of stimulation (active or sham stimulation) was administered. All the participants remained relaxed and rested during the stimulation. After the stimulation, the experimenter removed the tDCS equipment and then played a video that introduced basic financial statement analysis knowledge with the aim of helping the participants better understand the subsequent materials. Next, the participants were asked to sequentially complete the perceived investment potential measurement task, the equity sensitivity measurement task, and the risk preference measurement task. Finally, the socioeconomic features of the participants, such as their gender and investment experience (number of years of investment experience), were collected, and the experiment was over. Figure 2 shows the procedure of the experiment.

### Data Processing

The participants’ perceived investment potential was tested through three questions (Kelly et al., 2012; Kelly and Seow, 2016): (1) In your opinion, how attractive is Company X’s stock as a medium- to long-term investment? (2) In your opinion, what is the potential of Company X’s stock price to appreciate over the next 3 years? (3) In your opinion, what is Company X’s earnings potential over the next 3 years? All three questions were rated on −7 to +7 scales. This study took the average of the answers to the three questions as the participants’ perceived investment potential of Company X (Cronbach’s alpha = 0.87), denoted as *pip*. The mean value was 3.59, with a standard deviation of 2.55. Participants whose *pip* values were smaller than −4.06 (mean–3*s.d.) were regarded as outliers and were excluded from the following data analyses. Thus, 8 records (1 from the sham-medium treatment, 2 from the sham-high treatment, 3 from the active-medium treatment, and 2 from the active-high treatment) were excluded.

Equity sensitivity was evaluated by the participant’s total points from choices that placed more emphasis on one’s input in the five-item equity sensitivity instrument, denoted as *es*. The mean value was 22.24 (s.d. = 5.58), which was not far from but still significantly lower than the equity sensitivity of some US samples (Mueller and Clarke, 1998; Kickul and Lester, 2001) and Singaporean sample (Kelly and Seow, 2016) in previous studies (Mueller and Clarke: mean = 23.71, *t* = −2.42, *p* = 0.008; Kickul and Lester: mean = 25.15, *t* = −4.03, *p* < 0.001; Kelly and Seow: mean = 25.93, *t* = −4.45, *p* < 0.001).

The risk preferences of the participants were calculated based on Falk et al. (2018). The participants’ responses on the two parts of the risk preference measurement task provided two indicators for calculating their risk preference. These two indicators were standardized, multiplied by their weight, and finally summed. The sum was denoted as *rp*, and the mean value was 0.00 (s.d. = 0.83), which was not significantly different from the mean value of the Chinese participants in Falk et al. (2018) (mean = −0.07, *t* = 0.71, *p* = 0.480).

## Results

### The Effects of Transcranial Direct Current Stimulation

First, this study analyzed whether a high CEO-to-employee pay ratio reduced the participants’ perceived investment potential compared with the medium pay ratio treatments, regardless of whether the participants received sham or active stimulation. The *t*-test results show that there was a significant difference in *pip* between the participants from the high and medium pay-ratio treatments (mean: medium = 4.40, high = 3.61; *t* = 3.27, *p* = 0.001). These results indicate that in our experiment, the high CEO-to-employee pay ratio generally reduced the participants’ perceived investment potential in the construction company.

**FIGURE 3:**
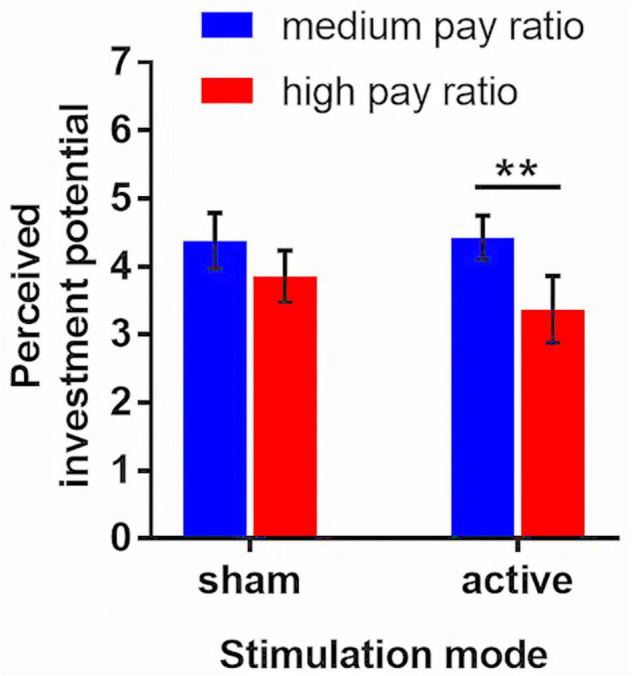
The means of the perceived investment potential in different treatments. The error bars indicate 95% confidence intervals. The asterisks indicate statistically significant differences (** 0.01). Sham-medium vs. sham-high: *p* = 0.751; active-medium vs. active-high: *p* = 0.014; sham-medium vs. active-medium: *p* = 1.000; sham-high vs. active-high: *p* = 0.938.

Next, this study examined how enhancing activity in the right TPJ affected the impacts of a high CEO-to-employee pay ratio on the participants’ perceived investment potential. One-way ANOVA was performed with *pip* as the dependent variable and *treatment* as the factor. Pairwise comparisons were performed, and Bonferroni correction was applied. The results show that the *pip* values were significantly different among the four treatments [*F*_(3,123)_ = 4.25, *p* = 0.006]. Specifically, there was a significant difference in *pip* between the participants from the active-medium treatment and those from the active-high treatment (mean: active-medium = 4.43, active-high = 3.37; *p* = 0.014). Nevertheless, there was no significant difference in *pip* between the participants from the sham-medium treatment and those from the sham-high treatment (mean: sham-medium = 4.38, sham-high = 3.86; *p* = 0.751). A comparison of the perceived investment potential under the four treatments is shown in Figure 3. Thus, enhancing activity in the right TPJ prompted the participants to differentiate between construction companies with high and medium CEO-to-employee pay ratios, with lower perceived investment potential for the former.

A regression was performed to test the robustness of the effects of tDCS, with *pip* as the dependent variable and *active* (active stimulation = 1, otherwise = 0), *high* (high pay ratio = 1, otherwise = 0), and their interaction active*high as the independent variables. The coefficient of *active* indicates the independent effect of receiving active stimulation compared with receiving sham stimulation when the CEO-to-employee pay ratio was medium. The coefficient of *high* indicates the independent effect of a high pay ratio compared to a medium pay ratio when the participants received sham stimulation. The coefficient of the interaction reflects the additional effect of receiving active stimulation on the difference in *pip* between the high and medium pay-ratio conditions compared to the effect of receiving sham stimulation on the difference in *pip* between the high and medium pay-ratio conditions.

Model 1 in Table 1 displays the result of the regression, which controlled for the participants’ risk preference *rp* and equity sensitivity *es*, the interaction es*high, and the perceived risk of investing in Company X (rated on 0–14 scales, with 0-very low risk and 14-very high risk), denoted as *prisk*. Unfortunately, the coefficient of the interaction active*high was non-significant, although its *p*-value was not far from a level of statistical significance (*p* = 0.138). In addition, a very significant negative effect of perceived risk on perceived investment potential was observed.

**TABLE 1:** OLS regressions on the effects of tDCS.

DV = *pip*	(1)	(2)	(3)	(4)	(5)
	Total	Females	Males	Inexperienced females	Inexperienced males
active	−0.079	0.344	−0.684	0.283	−0.417
	(0.321)	(0.500)	(0.411)	(0.633)	(0.556)
high	0.896	0.805	−0.010	2.432	−0.254
	(1.039)	(1.861)	(1.207)	(2.375)	(1.541)
active*high	−0.693	−1.242*	0.157	−2.557**	−0.106
	(0.465)	(0.735)	(0.586)	(0.925)	(0.774)
prisk	−0.213***	−0.105	−0.302***	−0.179*	−0.329***
	(0.045)	(0.075)	(0.052)	(0.099)	(0.066)
rp	0.180	0.198	0.157	0.332	0.195
	(0.138)	(0.214)	(0.175)	(0.247)	(0.215)
es	0.006	0.034	−0.031	−0.021	−0.036
	(0.029)	(0.050)	(0.035)	(0.058)	(0.043)
es*high	−0.045	−0.045	−0.012	−0.076	0.005
	(0.042)	(0.075)	(0.049)	(0.093)	(0.060)
Constant	5.484***	4.026**	7.086***	5.498***	7.122***
	(0.732)	(1.190)	(0.908)	(1.394)	(1.135)
*R* ^2^	0.204	0.146	0.358	0.282	0.419
Adj–*R*^2^	0.171	0.0710	0.302	0.185	0.336
*F*	6.168***	1.951*	6.377***	2.915*	5.055***
*N*	176	88	88	60	57
Achieved power	1.000	0.781	1.000	0.936	0.998

The dependent variable pip represents perceived investment potential; active = 1 if receiving active stimulation and=0 otherwise; high = 1 if the pay ratio is high and=0 otherwise; prisk represents perceived risk; rp represents risk preference; and es represents equity sensitivity. The standard errors are shown in parentheses. The asterisks indicate significant differences (* 0.1, ** 0.01, *** 0.001).

Furthermore, this study tested whether the effects of tDCS vary based on the participants’ gender and investment experience. Regressions were performed by gender, and the results are shown in model 2 and model 3 in Table 1. It was found that active*high was significant for the female participants, while *prisk* was significant for the male participants. Thus, there was a gender difference in the effect of both tDCS and perceived risk on perceived investment potential. Model 4 and model 5 in Table 1 further focus on participants with no investment experience, that is, participants who reported 0 years of investment experience. The effects of active*high on *pip* became much more significant for the inexperienced female participants (from *p* = 0.095 to *p* = 0.008), and *prisk* also became slightly significant. On the other hand, *prisk* remained the only significant independent variable for the inexperienced male participants.

### The Mechanisms Underlying the Effects of Transcranial Direct Current Stimulation

This subsection explores the mechanisms underlying the effects of tDCS. The results of the regressions performed are summarized in Table 2. Here, the study first took all female participants as the regression sample. As noted in the subsection on data processing, *pip* is the average of the answers to three questions regarding the attractiveness of Company X’s stock as a medium- to long-term investment (denoted as *stock_at*), the potential of Company X’s stock price to appreciate over the next 3 years (denoted as *stock_po*), and Company X’s earnings potential over the next 3 years (denoted as *earni_po*). Models 1–3 used the participants’ answers to each question as the dependent variables. The results show that the interaction active*high had a significant negative impact on *stock_po*, and perceived risk had a significant negative effect on *earni_po*. Model 4 further tested whether *prisk* was influenced by tDCS, and the answer was that it was not. Therefore, among the female participants, enhancing activity in the right TPJ decreased the perceived upside potential of the company’s stock only in the case of a high pay ratio.

**TABLE 2:** OLS regressions on the mechanisms underlying the effects of tDCS.

	(1)	(2)	(3)	(4)	(5)	(6)	(7)	(8)
Sample	Females				Inexperienced females			
DV	*stock_at*	*stock_po*	*earni_po*	*prisk*	*stock_at*	*stock_po*	*earni_po*	*prisk*
active	1.114	0.433	−0.514	−0.373	1.361	0.207	−0.718	1.419
	(0.824)	(0.597)	(0.486)	(0.744)	(1.094)	(0.803)	(0.575)	(0.853)
high	0.459	2.709	−0.754	0.287	1.878	3.363	2.055	5.464*
	(3.063)	(2.219)	(1.808)	(2.772)	(4.106)	(3.012)	(2.159)	(3.197)
active*high	−1.821	−1.746*	−0.158	−0.239	−3.490*	−2.920*	−1.260	−2.561*
	(1.210)	(0.877)	(0.714)	(1.095)	(1.599)	(1.173)	(0.841)	(1.229)
prisk	−0.067	−0.079	−0.168*		−0.155	−0.087	−0.294**	
	(0.123)	(0.089)	(0.072)		(0.172)	(0.126)	(0.090)	
rp	−0.203	0.773**	0.025	−0.114	−0.007	0.797*	0.205	0.222
	(0.353)	(0.256)	(0.208)	(0.319)	(0.428)	(0.314)	(0.225)	(0.341)
es	0.034	0.080	−0.011	−0.011	−0.054	0.034	−0.042	−0.019
	(0.082)	(0.059)	(0.048)	(0.074)	(0.100)	(0.074)	(0.053)	(0.080)
es*high	−0.020	−0.101	−0.013	0.048	−0.028	−0.106	−0.095	−0.085
	(0.124)	(0.090)	(0.073)	(0.112)	(0.160)	(0.118)	(0.084)	(0.128)
Constant	3.168	2.553*	6.357***	6.192***	5.186*	3.602*	7.706***	5.140**
	(1.959)	(1.420)	(1.156)	(1.634)	(2.410)	(1.768)	(1.267)	(1.793)
*R* ^2^	0.071	0.179	0.236	0.088	0.122	0.242	0.436	0.245
Adj−*R*^2^	−0.00978	0.107	0.169	0.0209	0.00388	0.140	0.360	0.160
*F*	0.880	2.485*	3.529**	1.310	1.033	2.368*	5.741***	2.866*
*N*	88	88	88	88	60	60	60	60
Achieved power	0.389	0.886	0.974	0.489	0.461	0.869	0.999	0.875

The dependent variables are as follows: stock_at represents the attractiveness of Company X’s stock as a medium- to long-term investment; stock_po represents the potential of Company X’s stock price to appreciate over the next 3 years; earni_po represents Company X’s earnings potential over the next 3 years; and prisk represents perceived risk. Active = 1 if receiving active stimulation and=0 otherwise; high = 1 if the pay ratio is high and=0 otherwise; rp represents risk preference; and es represents equity sensitivity. The standard errors are shown in parentheses. The asterisks indicate significant differences (* 0.1,** 0.01, *** 0.001).

Next, this subsection focuses on the sample of inexperienced female participants. The results of models 5–8 show that the interaction active*high had a significant negative impact on *stock_at* and *stock_po* but did not have a significant effect on *earni_po*. Nevertheless, perceived risk had a significant negative effect on *earni_po*, and *prisk* was significantly negatively influenced by the interaction active*high. The indirect effect of active*high on *earni_po* (through *prisk*) was significant (Sobel test: *p* = 0.080). Therefore, among the inexperienced female participants, on the one hand, enhancing activity in the right TPJ decreased the perceived attractiveness and upside potential of the company’s stock in the case of a high pay ratio; on the other hand, active stimulation reduced the perceived risk of investment in the case of a high pay ratio, which in turn increased the perceived earnings potential of the company. Nevertheless, the former effect seemed to be dominant in our experiment. Among the male participants or inexperienced male participants, although *stock_at*, *stock_po*, and *earni_po* were all significantly negatively influenced by *prisk*, the latter was not significantly related to tDCS.

## Discussion

This study applied tDCS to explore the role of the right TPJ in the effects of the CEO-to-employee pay ratio on potential investors’ perceived investment potential in the construction industry. The results show that enhancing activity in the right TPJ prompted only the female participants, especially those with no investment experience, to differentiate between construction companies with high and medium CEO-to-employee pay ratios, with a lower perceived investment potential for the former. For the female participants as a whole, enhancing activity in the right TPJ decreased the perceived upside potential of the company’s stock in the case of a high CEO-to-employee pay ratio. For the inexperienced female participants, enhancing activity in the right TPJ typically decreased the perceived attractiveness and upside potential of the company’s stock if it had a high CEO-to-employee pay ratio. Meanwhile, active stimulation reduced the perceived risk of investment in this case, which in turn increased the perceived earnings potential of the company.

The results obtained in the subgroup of inexperienced female participants were consistent with our prediction that a higher level of altruism may lead investors to be more aware of the negative effect of a high pay ratio on a company’s investment potential. However, these participants perceived that the negative effect mainly came from the stock market, as they decreased the ratings for the attractiveness and upside potential of the company’s stock. In other words, they speculated that other investors in the market would decrease their willingness to buy the company’s stock if a high CEO-to-employee pay ratio was disclosed. On the other hand, they might have believed that the high pay ratio was an indicator of high CEO attraction/retention ability (Kelly and Seow, 2016), reducing the financial risk of the company and thus increasing its earnings potential.

In our study, the effects of tDCS varied based on the participants’ gender and investment experience. Previous studies have demonstrated that females are more likely to make socially responsible investments (Tippet and Leung, 2001; Nilsson, 2008; Owen and Qian, 2008; Dorfleitner and Utz, 2014; Bauer and Smeets, 2015; Martin, 2019). This phenomenon may be partly because females have a lower perceived investment potential for a company with a low level of social responsibility (i.e., it has an unfair compensation structure), even if they have the same level of altruism as males. This effect may be more obvious for females with little or no investment experience. Nevertheless, as the experiment was performed in a university, it is difficult to recruit enough participants with investment experience and test whether the same effect exists for females with investment experience.

This study mainly focused on the construction industry by using a construction company in its experimental materials. The results here may be applied only to the construction industry. For instance, the inexperienced female participants increased their perceived earnings potential for the high-pay-ratio construction company after activity in the right TPJ was enhanced. However, disclosing a high pay ratio may lead to decreased perceived earnings potential for a company in the retail, service or other industries that face the mass market, as potential customers may reduce their spending on the products or services of the company due to its unfair compensation structure. Although this study tries to narrow its conclusions to the construction industry to avoid the problem of external validity, it may still provide valuable implications about the role of the right TPJ in investment decision-making regarding compensation in other industries.

This study also has other limitations. The participants were constrained to university students, who are different in many ways from real investors in the market. In addition, the study tested only the participants’ perceived investment potential, which does not guarantee their actual willingness or investment behaviors (Guo et al., 2022). Moreover, there may be other individual socioeconomic features in addition to gender and investment experience that can potentially affect the results. Future studies may compare the role of the right TPJ in investment decision-making regarding compensation between the construction industry and other industries or explore how the effects vary based on other individual features.

In conclusion, this study used tDCS to explore the role of the right TPJ in the effects of the CEO-to-employee pay ratio on potential investors’ perceived investment potential in the construction industry. The results show that enhancing activity in the right TPJ significantly reduced the perceived investment potential of female participants, especially those with no investment experience, when the company’s CEO-to-employee pay ratio was high compared to when the pay ratio was medium. The mechanisms underlying these effects of tDCS in the right TPJ on the perceived investment potential were also explored. The main contribution of this study lies in its pioneering exploration of the neural basis of investment decision-making regarding the CEO-to-employee pay ratio. Additionally, it reveals individual feature-based differences in the role of the TPJ in investment decision-making and its possible mechanisms.

## Data Availability Statement

The raw data supporting the conclusions of this article will be made available by the authors, without undue reservation.

## Ethics Statement

The studies involving human participants were reviewed and approved by Ethics Committee of the Key Laboratory of Applied Brain and Cognitive Sciences, Shanghai International Studies University. The patients/participants provided their written informed consent to participate in this study.

## Author Contributions

JW and SC performed the experiment and created the figures. All authors designed the experiment, analyzed the data, wrote and revised the manuscript, contributed to the article, and approved the version to be published.

## Conflict of Interest

The authors declare that the research was conducted in the absence of any commercial or financial relationships that could be construed as a potential conflict of interest.

## Publisher’s Note

All claims expressed in this article are solely those of the authors and do not necessarily represent those of their affiliated organizations, or those of the publisher, the editors and the reviewers. Any product that may be evaluated in this article, or claim that may be made by its manufacturer, is not guaranteed or endorsed by the publisher.
